# “Biodiversity Offsetting” in Uganda’s Protected Areas: A Pathway to Restoration of Forest Biodiversity?

**DOI:** 10.1007/s00267-024-01982-6

**Published:** 2024-05-10

**Authors:** Ritah Kigonya, Patrick Byakagaba, Edward Ssenyonjo, Charlotte Nakakaawa Jjunju

**Affiliations:** 1 Department of Geography, Norwegian University of Science and Technology, Trondheim, Norway; 2 Department of Environmental Management, Makerere University, Kampala, Uganda; 3 National Forestry Authority, Kampala, Uganda

**Keywords:** Biodiversity offsetting, Restoration, Forest composition, Forest structure, Livelihoods, Uganda

## Abstract

With limited national financing for conservation, there is an increasing interest in using biodiversity offset funds to strengthen protected area management. Offsetting measures can potentially be used in the restoration of degraded protected areas. However, there are concerns related to the uncertainty of restoration outcomes and time-lags before the expected benefits can be observed. Using a case of the Gangu Central Forest Reserve in central Uganda, we contribute empirical findings showing the potential and limitations of biodiversity offsetting by means of the restoration of a degraded forest reserve. We use forest cover change analysis and community surveys to determine forest changes after eight years of offset implementation, and forest inventories to analyse the current forest structure and composition to ascertain taxonomic diversity recovery. The results revealed that biodiversity offsetting led to a 21% increase in Tropical High Forest cover, and enhanced restoration of forest species composition and diversity. However, attaining permanence of the restoration benefits requires the regulation of community forest resource access and use. Strengthening forest management capacity to monitor the offset sites and compensating impacted communities for foregone forest resource benefits are crucial for the successful implementation of biodiversity offsets.

## Introduction

In the face of the current biodiversity extinction crisis, protected areas (PAs) are key for the conservation of biodiversity (Geldmann et al. 2019; Maxwell et al. 2020). The post 2020 Kunming-Montreal Global Biodiversity Framework suggested an increase in PAs so that 30% of land, sea and water is conserved (CBD 2022). However, effective PA management is constrained by a lack of resources, including funding, staffing and equipment (Leverington et al. 2010; Watson et al. 2014). Biodiversity offsetting is promoted among innovative ways to create new conservation areas, to restore degraded ecosystems, or to fund the improved management of already existing protected areas (Pilgrim and Bennun 2014; Maron et al. 2015; Githiru et al. 2015). The measure seeks to compensate for loss of biodiversity in one place with equivalent gains elsewhere (BBOP 2009). Biodiversity offsetting is carried out as the last option of what is often described as the ‘mitigation hierarchy’, after avoidance, minimization and restoration measures have been implemented, in order to fully compensate for the residual environmental impacts of development activities to achieve no net loss (NNL) benefits (BBOP 2009).

Restoration of degraded lands, both within and outside existing protected areas, is among the major measures of biodiversity offsetting (Maron et al. 2012; Moilanen and Kotiaho 2018; Simmonds et al. 2020). By 2018, 18.8% of all documented offset projects were ecological restoration projects, and an additional 46.4% combined ecological restoration and avoided loss measures (Bull and Strange 2018). However, ascertaining the effectiveness of restoration biodiversity offsets (BOs) in attaining the desired conservation benefits, as well as of the conditions supporting the achievement of conservation outcomes has not attracted much research attention. Such assessments can guide the effective implementation of BOs and the development of related evidence-based policy (Kormos et al. 2014; Bull and Strange 2018).

The potential of such BO measures has been discussed by some scholars, and deemed to be feasible and useful in securing existing PAs when their implementation: strictly follows the biodiversity mitigation hierarchy; involves designing appropriate baselines, metrices and guidelines that can be used to evidence achievement of NNL benefits; secures permanence of biodiversity NNL benefits; prevents social inequity; and safeguards social wellbeing (Githiru et al. 2015; Buschke et al. 2019). However, offsetting in protected areas presents major concerns about the risks of cost-shifting, whereby biodiversity funds displace rather than supplement current or future conservation-funding commitments. In such a case, biodiversity benefits would not be additional.

There are challenges in achieving equivalence in biodiversity losses and gains, as the prioritization of offsetting in protected areas involves the risk that biodiversity in the development and offset sites will not be comparable. There is uncertainty with regard to ensuring the permanence of biodiversity benefits, whereby offset gains last for as long as the development impacts. Offsetting in protected areas can lead to negative social impacts, especially where the destruction of a given ecosystem upon which a community is dependent is compensated for by the restoration or protection of another ecosystem, or if the offset site is distant from the affected community, limiting their access to the offset site, or if community access to the protected area resources is restricted (Pilgrim and Bennun 2014; Githiru et al. 2015).

For a better understanding of the potential and limitations of restoration BO in achieving desired protected area conservation benefits, more empirical studies are needed. Using a case of Gangu Central Forest Reserve (CFR) in Central Uganda, we ascertain whether BO can contribute to restoration of degraded protected forests. The Paper: (i) analyses the forest cover changes to determine forest gain after 8 years of offset implementation; (ii) analyses the current forest structure and composition to ascertain forest taxonomic diversity recovery; (iii) investigates the reasons for changes in the status of the BO sites as well as community dependence on forest resources prior to and after the implementation of BO; (iv) and provides an insight into factors that support the achievement of restoration conservation benefits, which may support biodiversity policy and implementation.

## Materials and Methods

### Study Area

#### Gangu Central Forest Reserve

**Fig. 1:**
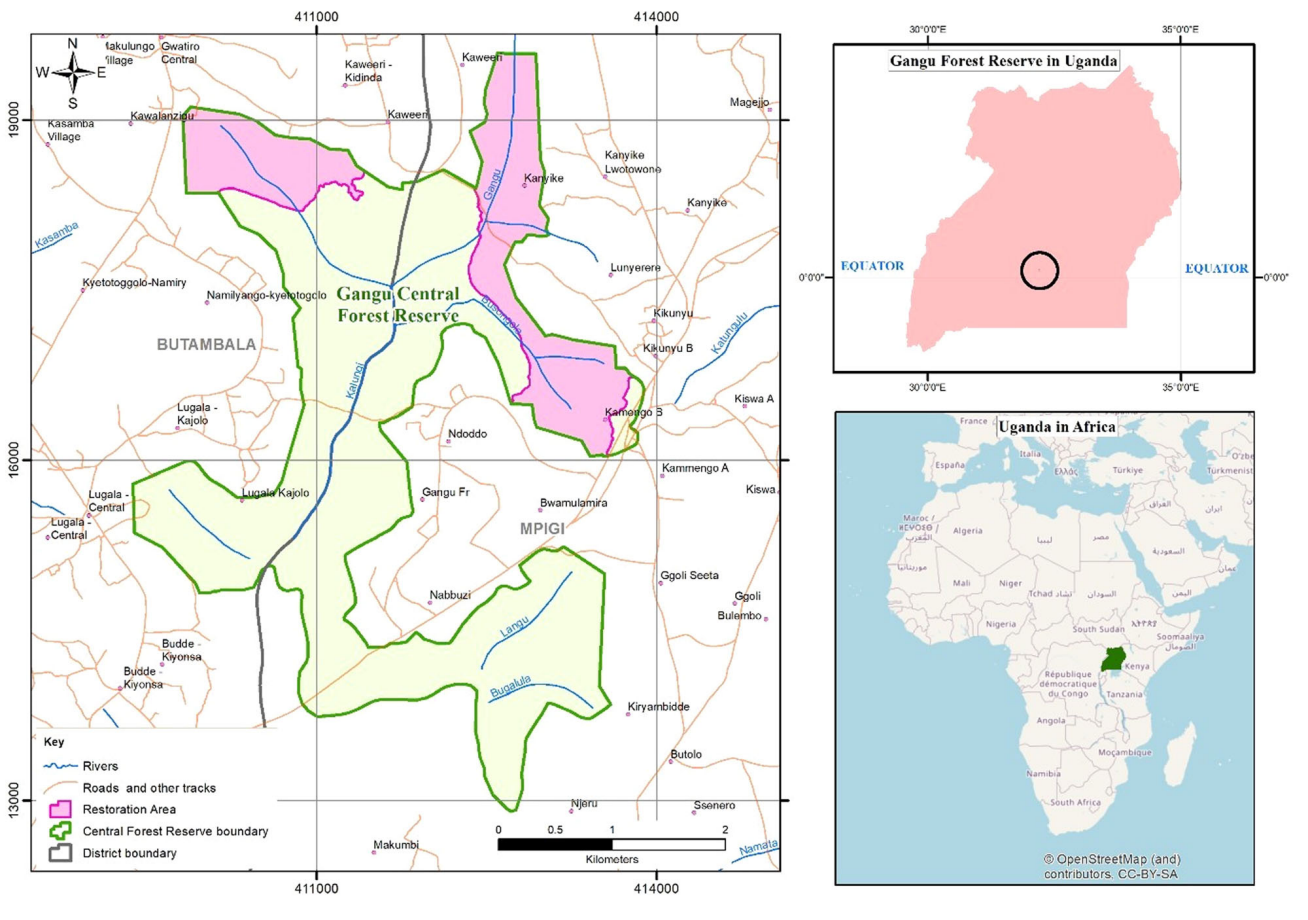
Map of the Gangu Central Forest Reserve showing the biodiversity offset sites and villages neighbouring the forest

Gangu CFR is located on the western shores of Lake Victoria, in Mpigi and Butambala districts of Central Uganda (Fig. 1). The whole forest reserve is classified as a Strict Nature Reserve (SNR) (MWLE 2002). In Uganda, all forest reserves are managed in accordance with the sustainable forest management principles which include the conservation of ecosystems, habitats and biological diversity; sustaining the potential yield of the ecological, social and economic benefits of forests; and the improvement of livelihoods and reduction of poverty (GoU 2003). The forests are zoned into three management areas, namely; strict nature reserves, buffer zones and production zones. The whole forest is managed under the same objectives (for ecological, forestry and tourism purposes), with the distinction between zones characterised by a shift of emphasis within the set of objectives, rather than the definition of a completely different objective for each zone (MWLE 2002). As such, forest zones are based on utilization or management purpose and not biodiversity. There are more restrictions on forest resource extraction within the SNR, followed by the buffer zones and the highest levels of forest extraction and utilization in the production zones. When compared to the IUCN categories of protected areas, forest reserves fall in category VI (protected area with sustainable use of natural resources). According to the Uganda Forestry Nature Conservation Master Plan (2002), Gangu CFR was proposed as a SNR because it was relatively intact with encroached areas successfully restored.

Gangu CFR covers 11 km^2^ and was historically dominated by a tropical high forest community classified as Type C2 (*Piptadeniastrum*-*Albizia*-*Celtis* forest) (MWLE 2002). By 2010, the reserve – together with other small reserves forming the Mpigi group of forests—was reported to be heavily degraded due to illegal commercial firewood cutting, illegal logging, charcoal burning and encroachment for agricultural expansion (SMEC International Pty Ltd 2010). The forest is easily accessible, as part of it is traversed by a major highway (Kampala-Masaka Road) that connects the capital city Kampala to the western region (MWLE 2002). The forest is managed by the National Forest Authority (NFA), an autonomous agency with the mandate to manage Central Forest Reserves (CFRs) in Uganda (GoU 2003). However, among the constraints to NFA’s operationalization of the protection and management of CFRs is inadequate financial resources and staffing (NFA 2016a; MWE 2020). The institution obtains funds through government financing, Non-Tax Revenue and donor financing which has greatly reduced over the years (NFA 2016a). Public funding, which is the major source of funds for NFA is limited mostly to covering salaries and wages, with very little left to meet the operational expenses for law enforcement, boundary mapping and the restoration of degraded reserves (MWE 2016). According to the Biodiversity Expenditure Review (BER) for the Ministry of Water and Environment for the fiscal period 2005/6 to 2014/15, only 6% of the Water and Environment sectoral budget had facilitated biodiversity management (NEMA 2017). In some instances, planned activities were not implemented or completed, due to limited budget releases (MWE, 2016).

#### Biodiversity offset in the Gangu Central Forest Reserve

The Gangu forest reserve BO was established in 2014 to compensate for vegetation cleared in seven forest reserves (covering a total of 27.12 ha) (Appendix 1) during the establishment of the Kawanda-Masaka electricity transmission line (Katusabe 2017). According to the Environmental and Social Impact Assessment (SMEC International Pty Ltd 2010), the forest reserves were degraded, constituting small sized trees that merged into bushes. Some of the reserves were small patches that bordered swamps and heavily modified by encroachment for agriculture, brick making and fuel wood. All the reserves are located in two neighbouring districts of Mpigi and Butambala on the western shore of Lake Victoria, between Latitude 0^o^0 and 0^o^3N and between Longitude 21^o^45 and 32^o^30E. The line was constructed to transport electricity generated from the Bujagali hydro-power plant in Kampala (the capital city of Uganda) to the western region. The government of Uganda, via the NFA, implemented the project using financial aid borrowed from the World Bank (World Bank 2021). This was in collaboration with the Uganda Electricity Telecommunication Company Limited (UETCL). The BO was implemented to restore forest cover on 200 ha of degraded forest. According to the implementation, completion and results report for the development project (World Bank 2018), a second BO was established for the restoration of 10 ha of Nabijoka Local Forest Reserve (LFR). However, the implementation of the second BO was not a focus of this study. At the time of development, project approval and implementation in 2011, Uganda had no provisions for BO implementation within its regulatory or policy frameworks. The BO was thus implemented to ensure compliance with the World Bank safeguard policy OP/BP 4.36 (Forests) (World Bank 2002).

The operationalization of the BO included boundary opening and marking of the whole forest reserve, increased patrols, restricting forest access and resource use to the collection of firewood from fallen tree branches, prohibiting logging activities, and the eviction of ‘encroachers’ who were either grazing animals in the forest or practicing what Cavanagh and Benjaminsen (2015) termed ‘guerrilla agriculture’ – i.e., strategic crop production by farmers circumventing forest regulations enacted by the state to reserve areas for nature conservation.

Prior to commencing the offset activities, the ‘guerrilla farmers’(farmers stealthily growing crops in the reserve) were informed about the upcoming restoration activities in the forest. To secure community acceptance and support for the offset implementation, the NFA initiated Collaborative Forest Management (CFM) arrangements with the ‘guerrilla farmers’. Accordingly, the ‘guerrilla farmers’ were allocated portions of the Gangu CFR land classified as CFM areas for commercial tree planting for a period of ten years. The farmers were only permitted to plant fast-growing eucalyptus trees, for the quick production of firewood, poles and timber, and the generation of income through the sale of tree products. This was meant to enable forest farmers to build up capital to start forest-independent livelihood activities. In return, the farmers were expected to stop cultivating in the forest, and to support the NFA in patrolling and monitoring the BO sites for illegal activities.

The eviction of these farmers from the BO sites was followed by restoration or enrichment planting (with indigenous tree species) in areas that had previously been used for subsistence farming. This was followed by spot weeding and the liberation of planted seedlings to facilitate natural regeneration. Planting activities commenced in 2014, when approximately 100 ha of the forest was planted with native tree species (NFA 2015). In 2015, an additional approximately 100 ha of the reserve area was planted (NFA, 2016b), bringing the total amount of replanted forest to approximately 200 ha (World Bank, 2021) (Fig. 1). In 2019, the restoration of an additional 50 ha of degraded forest land adjacent to the electricity transmission powerline wayleave commenced as an extension to the BO. According to NFA officials, the extension aimed to compensate for forest cover that was lost due to increased forest access facilitated by the establishment of the transmission line. By the time of the study, these trees were still very young and were therefore not considered during the assessment. Seven tree species were planted during the restoration of the offset sites: *Terminalia superba, Terminalia ivorensis*, Bathedavia, *Khaya anthotheca*, *Prunus africana*, *Maesopsis eminii* and *Cedrela odorata*. UETCL financed the implementation of the biodiversity offset activities through a one-off payment to the NFA that was determined by the cost of restoration and management of the restoration area as prescribed in the Memorandum of Understanding. The NFA was obligated to ensure that the area is restored into a forest using the payment made to them as a form of compensation. Maintenance of the biodiversity offset area was left to the NFA as per the Memorandum of Understanding between the NFA and UETCL. This was because the sites remained part of a central forest reserve despite being a BO. The NFA is responsible for the management of all central forest reserves (GoU 2003).

The design and implementation of offset schemes involves loss-gain accounting to verify that biodiversity losses and benefits are equivalent (Bull et al. 2013). We sought information about the offset design and implementation from UETCL and the NFA, but did not receive any documentation despite all enquiries being made through the known procedures. In the Environmental and Social Impact Assessment Report that we obtained (SMEC International Pty Ltd, 2010), there was no mention of baseline studies prior to the implementation of the offset to quantify the residual loss against which biodiversity benefits would be compared. Therefore, there was no frame of reference for the offset including counterfactual scenarios for the biodiversity status of the reserve. We did not come across stipulated requirements for NNL of biodiversity. There were no attempts to attain equivalence in species, composition, structure, and functionality between the development and offset sites during BO design. In addition, we had no access to the data that is required in order to monitor and evaluate the ecological effectiveness of the BO. Such data often includes: the amount and types of gains required/expected from BO actions, monitoring data for target biodiversity at the offset site to verify gains, and monitoring data at the impact and control sites to test counterfactual assumptions (Kujala et al., 2022). In that respect, the project falls short of the requirements of an offset and should have been referred to as an ecological compensation (BBOP 2009).Due to the data gaps, this paper does not assess NNL and additionality that encompasses biodiversity benefits attained exclusively due to the implementation of offset activities. Nevertheless, recent suggestions are that biodiversity offsetting should go beyond achieving relative gains against a counterfactual scenario of biodiversity decline at the project level, and aim at achieving absolute gains at jurisdiction levels so as to achieve broader conservation goals (Simmonds et al. 2020). Even with no focus on achieving project level NNL benefits, restoration followed by protection leads to an increase in biodiversity features that is essential for achieving jurisdictional-level No Net Loss that is greatly desired (Simmonds et al. 2020).

Therefore, the analysis of forest characteristics was made in reference to what the offset sites could have constituted prior to its degradation through human activities. The study considered the 7.04 ha of forest land cleared during the establishment of the wayleave as the impacted area within the Gangu Forest Reserve, and the 200 ha restoration area through biodiversity offset financing as the offset area.

### Research Design, Methods and Sampling Strategy

The study applied remote sensing (RS) to detect forest cover changes, with an analysis of forest inventory and social survey data to obtain qualitative and quantitative information about changes in forest cover, composition and structure eight years after the offset implementation.

#### Forest Cover Change Analysis

We mapped the offset areas using a hand-held GPS device in October 2021. We acquired satellite images taken at three different dates from Landsat 8 (2013) and Sentinel 2 (2018 and 2022). We preferred Sentinel 2 due to its higher resolution, but Sentinel imagery was not available in 2013(launched in 2015). We thus combined the more recent Sentinel 2 images with the Landsat image from 2013.

The images were acquired from the United States Geological Survey (USGS) via the Earth Explorer web portal (www.glovis.usgs.gov). For each of the selected years, we aimed to obtain images from the same season/month. However, this was not possible due to cloud cover. We therefore used cloud-free satellite images from different seasons of the selected years – namely, July 2013, September 2018 and January 2022. Located close to the equator, the Gangu CFR experiences minimal variations in vegetation cover in the different seasons or months of a year. This is because regions closest to the equator receive large amounts of rainfall all-year round, which means that they experience very little change in ambient temperature or relative humidity throughout the year (Chan et al. 2002; Machado et al. 2004).

The images were processed to provide surface reflectance values by removing atmospheric effects in order to mitigate the impact of seasonality and to make objects in the images as clear as possible. Several bands of each satellite image were stacked into multiband composites to be used for the classification and analysis. For the Landsat 8 image, six bands from 2 to 7 were stacked. For Sentinel 2 images, four bands (2, 3, 4 and 8) were stacked. Object-based image analysis was applied to images using the Mean Shift filter in the Orfeo Toolbox, which segmented them into distinct objects based on their signatures (https://www.orfeo-toolbox.org/). The Random Forest algorithm in the R software (https://www.r-project.org/) was used to classify each object to the respective predetermined class.

Random Forest is a non-parametric supervised learning algorithm that can identify and cover classes with high accuracy, despite subtle differences due to seasonality. Therefore, the use of a combination of satellite images processed to provide surface reflectance together with the capabilities of the Random Forest algorithm mitigated the impact of the subtle seasonal changes that may have occurred in the study area.

The pre-determined classes were: (1) plantation and woodlots, (2) tropical high forest (THF) – normal stock, (3) tropical high forest (THF) – low stock, (4) bushland, (5) wetlands, (6) subsistence farming, and (7) built-up area. As chlorophyll reflects near-infrared light (NIR), false-colour composites were used for expert visual validation of the image classifications—i.e., bands 5 (near-infrared), 4 (red) and 3 (green) of the Landsat image, and bands 8 (visible and near infrared (VNIR)), 4 (red) and 3 (green).

#### Forest Inventory

We sampled 83 plots of 20 × 20 m (a total area of 3.32 hectares), which were selected systematically, 100 m apart from each other, along transects in the offset sites. Given the nature of the Gangu CFR as a meandering forest, with swampy areas in the middle, the starting points for the transects were purposively laid 100 m from the forest edge to avoid the sampling of disturbed forest along the forest edge or of swampy areas in the central parts of each forest arm. Swampy areas were avoided because they did not undergo forest restoration. Given a total BO area of 270.3 ha forest restoration area, the sampling intensity was 1.2%. Within the plots, tree diameter at breast height (1.3 m) was measured using a diameter tape. All trees with a diameter at breast height (DBH) that was greater than or equal to 5 cm were identified and recorded. Trees with buttresses were measured above the buttresses. Trees that could not be identified were classified under one family, named ‘unknown’. Tree heights were measured using a Suunto clinometer. A 2 × 2 m (0.0004 hectare) subplot was established in each plot, in which seedlings/resprouts with a collar diameter of less than 5 cm were identified and counted. Plots undergoing cultivation and grazing were also recorded.

#### Social Data

Household interviews, key informant interviews and Focus Group Discussions (FGDs) were conducted to acquire information on the trends and cause(s) of forest cover changes, on forest state, resource access and use prior to and after implementation of the offset, and on community engagement in offset activities. Obtaining this information was crucial because no baseline biodiversity or socio-economic data prior to implementation of the offset were readily available from the responsible entities. One village was randomly selected from each parish adjacent to the Gangu CFR, making a total of 6 villages. A household survey was conducted by means of convenience sampling of 140 respondents who had spent a minimum of eight years in the villages—namely, Kiryambidde (32), Kanyike (25), Makulungo (25), Kajoolo (20), Budde Kiyonsa (19) and Ndoddo (19) (Fig. 1). Eleven key informant interviews were conducted with nine forest-dependent ‘guerrilla farmers’, one charcoal producer and one brick layer. The respondents were either recommended by the local council leaders or identified during household interviews. In addition, CFM members, local council leaders, and representatives of forest management authorities were also interviewed. FGDs were held in two villages neighbouring the offset sites: in Ndoddo (10 men and 9 women, separately) and in Kanyike (6 men and 4 women in one group). FGD participants were purposively selected from a list of members that had lived in the villages for more than 8 years.

### Data Analysis

#### Forest Cover Change Analysis

Using the Orfeo Toolbox (OTB), a software library for processing images from Earth observation satellites, the satellite images were processed in three steps to produce land use/cover maps: (1) feature extraction; (2) selection of training data (signatures); and (3) adaptation of the National Biomass Study (NBS) classification system. The image classification was guided by field reconnaissance information gathered in August 2022. Out of the 13 classes on the NBS system, 4 land use/cover classes were identified and mapped: THF-normal stock, THF- low stock, bushland, and subsistence farmland (Table 1). Inventory data from the 83 plots were used for classification accuracy assessment. The resulting three land use/cover maps (2013, 2018 and 2022) were compared quantitatively using a change matrix.

**Table 1:** Land use/cover classifications mapped in the Gangu biodiversity offset sites

Land use/cover classification	Definition
Tropical High Forest (THF)-normal stock	These are natural forests rich in species biodiversity i.e. flora and fauna
Tropical High Forest (THF)-low stock	Encroached or recovering natural forest with reduced species richness and composition dominated by secondary growth of bush and shrubs.
Bushland	Comprises bush, thickets, scrub (average height < 4 m)
Subsistence farming	Comprises of mixed farmland, small holdings in use or recently used, with or without trees

Source: National Biomass Study Technical Report 2003

#### Forest structure and composition

The data was entered into Microsoft Excel to create data files and sorted using pivot tables. The species identified within the sampled plots were counted to determine species composition and richness. Species dominance was obtained by calculating relative species density (Savadogo et al. 2007) using the following equation:

1 $${RD}=\frac{{Number\; of\; individuals\; of\; one\; species}}{{Total\; Number\; of\; all\; individuals\; in\; all\; species\; encountered}}x100$$

Species diversity was determined by calculating the Shannon Diversity index:

2 $$H=-\sum \left[\left(p{\rm{i}}\right)\times \log \left(p{\rm{i}}\right)\right],{\rm{where}}\,{p}_{i}=n/N$$

where:H- Shannon diversity index; p_i_- proportion of individuals of i-th species in a whole community; ∑- sum symbol; log- the natural logarithm; n– total number of individuals of a given species; and N- total number of individuals in a community.

The Shannon Diversity index was derived using SDR 4 software (Seaby and Henderson 2006). Population structure was analysed based on the diameter class distributions.

#### Social Data

The survey data was analysed using the Statistical Package for Social Sciences (SPSS), a software package used for the analysis of statistical data. Descriptive statistics were run to obtain summaries on socio-demographic characteristics of respondents, respondents’ perceptions on forest status and change before and after the implementation of the BO, the reasons for the changes, and changes in forest resource access and use. Key informant interviews, and FGDs were recorded, transcribed and thematically analysed. Narratives of different social categories of respondents were mapped from the content under each identified theme from the transcriptions including forest status, use, access and change.

## Results

### Forest Cover Changes in the Offset Sites

**Fig. 2:**
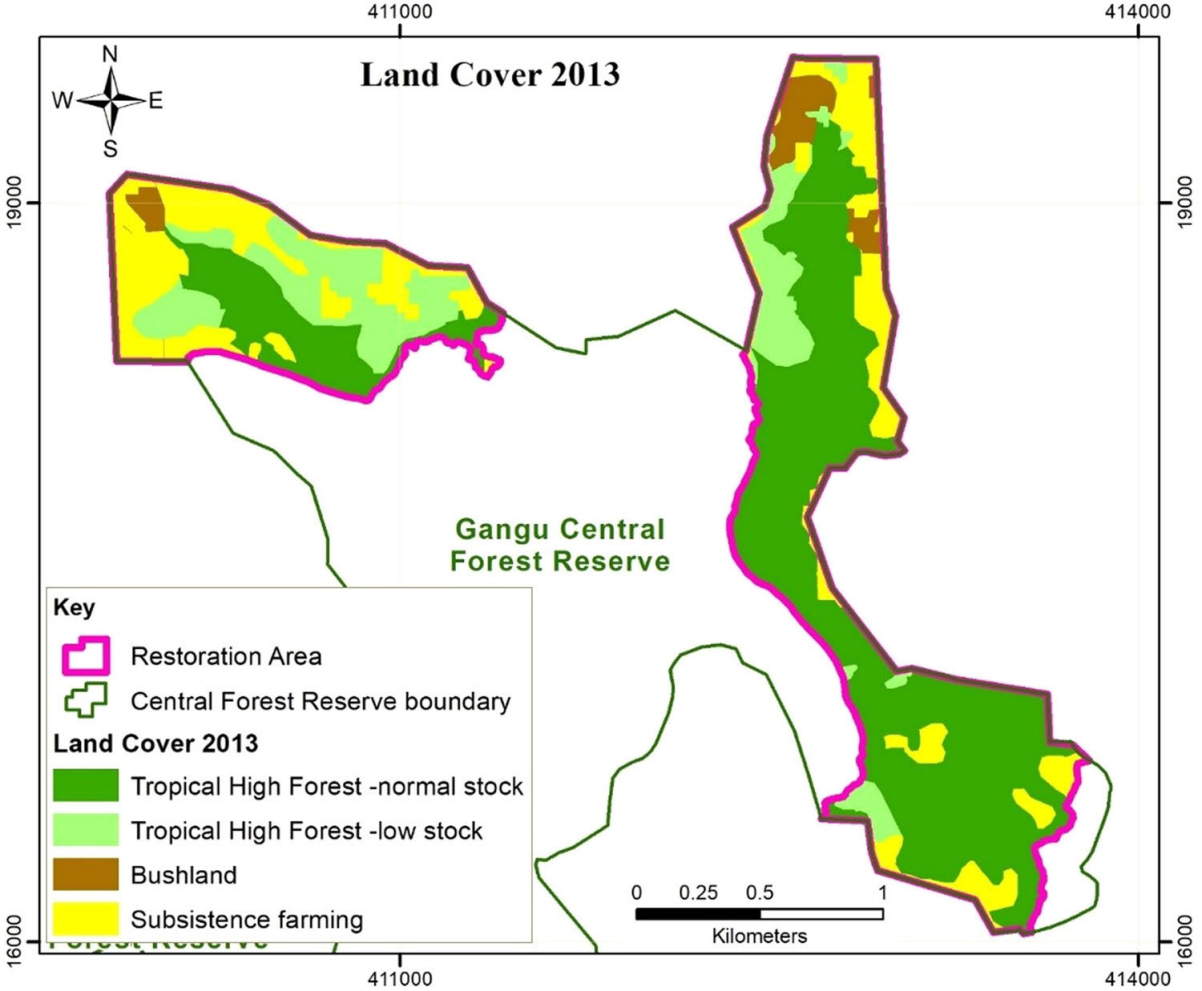
Land use/cover in the Gangu biodiversity offset sites in 2013

**Fig. 3:**
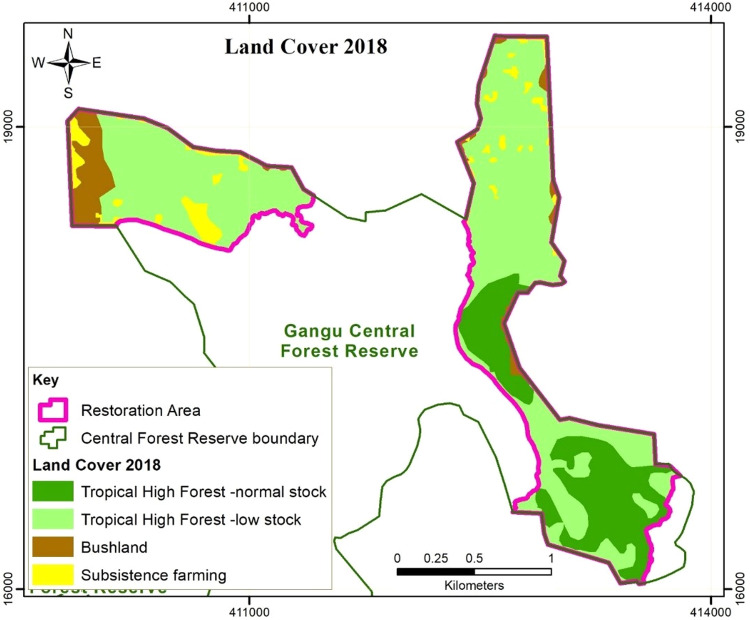
Land use/cover in the Gangu biodiversity offset sites in 2018

**Fig. 4:**
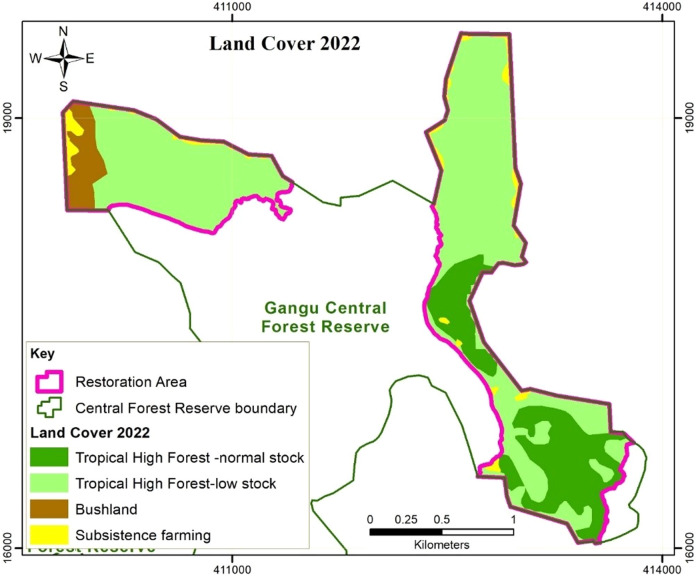
Land use/cover in the Gangu biodiversity offset sites in 2022

The satellite image analyses (Figs. 2–4) revealed a decline in the areas under THF-normal stock and subsistence farming, and an increase in the areas under THF-low stock and Bushland within the BO sites. In 2013, prior to the implementation of the restoration activities, 55%, 17% and 25% of the offset sites was THF-normal stock, THF-low stock and subsistence farmland respectively. Within 8 years after the onset of restoration activities, the total area covered by THF-normal stock significantly decreased to 21%. Out of the 147.7 ha of THF-normal stock in 2013, 62.9% was converted to THF-low stock in 2022 through a reduction in the numbers of big trees. Some of the trees planted in the frame of the restoration activities were also found cut down during the data collection for the forest inventory in 2022.

Total area under subsistence farming decreased to 5%, through regeneration of 72% and 13.8% of its proportion to THF-low stock and bushland respectively. Area under THF-low stock increased to 69% of the offset sites, with 49.5% of the area acquired through degradation of the normal stock THF, 4.2% and 25.6% was acquired through regeneration of the bushland and farmed land respectively, while 20.8% had not changed. Although the area under subsistence farming decreased by 82%, there were still a few scattered gardens as shown in Table 2. The gardens were either freshly cleared within the forest, or were previously existing prior to offset implementation. We observed that some of the farmers planted shade tolerant crops such as coffee and bananas. The trees were widely scattered in the farms to avoid canopy closure that would constrain crop growth. Such farmers have maintained the trees planted during the restoration exercise, while clearing new seedlings and resprouts.

**Table 2:** Land Use Change matrix in the Gangu biodiversity offset sites from 2013 to 2022

2013 to 2018					
Land use/cover 2013 (ha) (initial)	Land use/cover -2018 (ha) (final)				Total area 2013
	THF-normal stock	THF-low stock	Bushland	Subsistence farming	
THF-normal stock	54.7	88.4		4.6	147.7
THF-low stock	2.5	39.2	2.2	2.0	45.9
Bushland		7.3	2.0	0.7	10.0
Subsistence farming		44.0	13.8	9.0	66.7
Total area 2018	57.2	178.9	17.9	16.3	270.3

2018 to 2022					
Land use/cover-2018 (ha) (initial)	Land use/cover-2022 (ha) (final)				Total area 2018
	THF-normal stock	THF-low stock	Bushland	Subsistence farming	
THF-normal stock	56.8			0.4	57.2
THF -low stock		176.8		2.1	178.9
Bushland		1.8	13.1	3.0	17.9
Subsistence farming		9.3	0.3	6.8	16.3
Total area 2022	56.8	187.8	13.4	12.3	270.3

2013 to 2022					
Land use/cover-2013 (ha) (initial)	Land use/cover-2022 (ha) (final)				Total area 2013
	THF-normal stock	THF-low stock	Bushland	Subsistence farming	
THF-normal stock	54.3	92.9		0.5	147.7
THF-low stock	2.5	39.1	2.2	2.2	45.9
Bushland		7.8	2.0	0.2	10.0
Subsistence farming		48.0	9.2	9.5	66.7
Total area 2022	56.8	187.8	13.4	12.3	270.3

The results from the household survey show that the majority of the respondents (74%) reported a decrease in forest cover prior to the commencement of BO restoration activities in 2014, while 21% and only 3% reported no change or an increase in forest cover respectively. The remaining 2% indicated limited knowledge about the status of the forest. The responses did not vary significantly across respondents’ social and demographic characteristics. Respondents reported logging (100%), forest farming (72%) and weak law enforcement (70%) as the major drivers of forest loss. The other drivers of forest loss included increased forest dependents (23%), grazing (9%) and establishment of forest plantations (7%). According to the FGDs and key informant interviews, most of the forest cover loss was witnessed in the period 2010–2016. Extensive logging activities were driven by high demand for forest products including timber, charcoal and firewood in the neighbouring urban centres. Extraction of the big trees created forest openings which the local communities turned into farm lands to cultivate ginger, the main cash crop in the communities adjacent to the forest. Some of those who were engaged in forest farming reported having had to make seasonal payments in form of bribes to the forest rangers and forest police to maintain their activities in the forest. As such, forest officials were considered key facilitators of forest degradation.

**Fig. 5:**
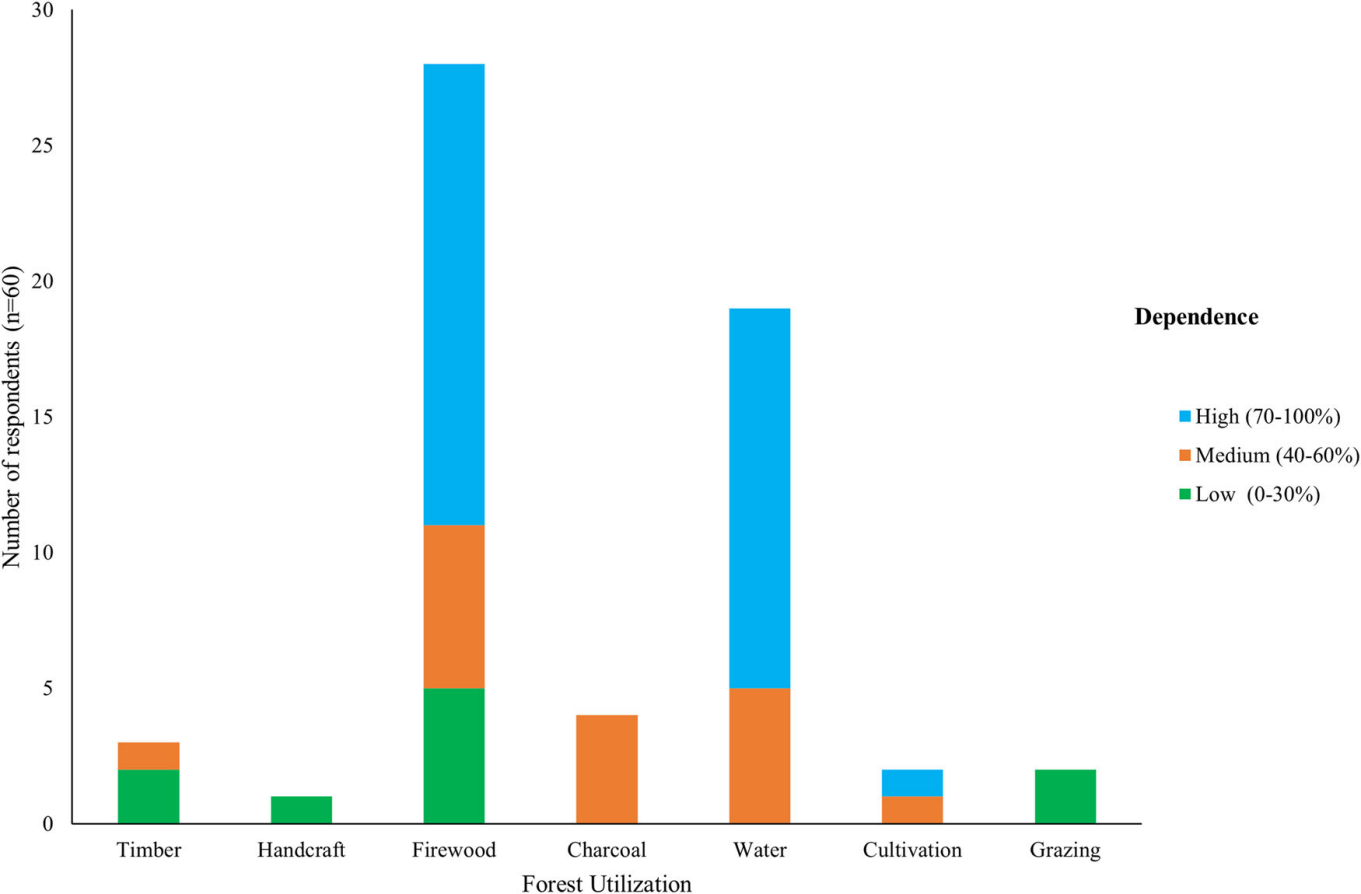
Forest utilization activities in Gangu biodiversity offset sites and level of community dependence on the activities

Between 2014 and 2022, 54.7% of the survey respondents reported to have observed an increase in the forest cover of the BO sites, whereas 12.5% and 29.7% reported no change and a decline in forest cover respectively, while 3.1% indicated limited knowledge about the status of the offset sites. Increase in forest cover was attributed to restoration of the degraded forest areas, increased restrictions on access and use of forest resources in the offset sites and forest patrol support from the CFM members. Communities were only permitted to collect firewood, water, and handcraft materials. However, some activities that were not permitted such as logging for timber and charcoal, farming and grazing were also still carried out by a few individuals as shown in Fig. 5. We also found goats and cows browsing and grazing in the forest during field work activities. Respondents that reported a decline in the forest cover attributed it to continued logging and farming practices, and weak law enforcement resulting from corruption among forest officials. These claim the remnants of the forest farms are sustained through bribing forest rangers and forest police. Logging activities are usually carried out in the middle of the night. According to FGDs, illegal logging was mainly carried out to obtain fuelwood, especially charcoal. During forest inventories, we came across cut trees, including those planted during the restoration activities. Key informant interviews with forest authorities revealed that, to reduce pressure on the offset sites, they established CFM sites outside the BO sites, and allocated the forest land to individuals previously cultivating in the offset sites to establish commercial tree plantations. These were intended to provide alternative sources of timber, poles, charcoal and firewood to the forest adjacent communities as well as an alternative source of income to forest farming. It was thus a form of compensation for lost livelihood benefits due to restrictions on forest resource access and use in the offset sites.

However, the beneficiaries were very few, approximately 60 individuals, leaving the majority of the forest dependents without alternative sources of forest products and livelihoods.

### Forest Composition and Structure

#### Species Composition and Richness

A total of 1318 individual trees of DBH equal to or greater than 5 cm were recorded in 82 (20 × 20 m) of the 83 sampled plots within the BO sites. There were no trees in one sample plot. The trees recorded comprised of 62 species, 52 genera, and 29 families (Appendix 2).

The most dominant families were Moraceae, followed by Meliaceae, Rhamnaceae, Combretaceae, and Anacardiaceae. The most dominant genera were Maesopsis, followed by Cedrela, Terminalia, Pseudospondias, and Ficus. Based on relative density, the most dominant tree species were *Maesopsis eminii* (13.2%), *Cedrela odorata* (9.6%), *Pseudospondias macrocarpa* (9.2%), *Terminalia superba* (8.4%), and *Antiaris africana* (5.4%).

The least abundant tree species (each with a percentage of 0.1) were *Albizia coriaria*, *Alconia sp*, *Bridelia micrantha*, *Fagara macrophylla*, *Harungana madagascariensis*, *Prunus africana*, *Teclea nobilis*, and *Tetrapleura tetraptera*. Seven of the tree species in the offset sites were on the IUCN Red List of threatened species, 8 were singletons (of which 1 was an unknown species), that is species that have only been observed once (Lim et al. 2012), while 19 tree species are listed among the nationally reserved/ protected tree species in Uganda (Table 3). Using Species Diversity and Richness (SDR) 4 software, we obtained a Shannon diversity index of 3.312 and Pieulous J evenness of 0.8025.

**Table 3:** Dominant species, families, and genera; IUCN Red Listed species, nationally reserved species and singletons within Gangu biodiversity offset sites (numbers in brackets indicate the sum of attributes from all plots)

Dominant Species > 5 cm dbh	Dominant Families	Dominant Genera	IUCN Red Listed	Nationally Reserved	Singletons
*Maesopsis eminii* (174)- Colonizer	Moraceae (198)	Maesopsis (174)	*Entandrophragma utile* (12)	*Albizia coriaria*	*Tetraplaura. tetraptera*
*Cedrela odorata* (127)- (Colonizer)	Meliaceae (188)	Cedrela (127)	*Fagaropsis angolensis* (6)	*Albizia grandibracteata*	*Alchornea sp*
*Pseudospondias macrocarpa* (121)*-* Canopy of established forest	Rhamnaceae (174)	Terminalia (127)	*Khaya anthotheca* (18)	*Albizia zygia*	*Bridelia micrantha*
*Terminalia superba* (111)- Colonizer	Combretaceae (127)	Pseudospondias (121)	*Lovoa trichilioides* (10)	*Aningeria altissima*	*Fagara macrophylla*
*Antiaris africana (71)-* (canopy of regenerating forest)	Anacardiaceae (123)	Ficus (104)	*Milicia exelsa* (4)	*Canarium schweinfurthii*	*Harungana madagascariens*
*Trema orientalis (65)-* Secondary plant succession (pioneer species)	Ulmaceae (107)	Antiaris (71)	*Olea welwitschii* (5)	*Entandrophragma utile*	*Prunus africana*
*Ficus sur* (59)- Colonizer	Cornaceae (54)	Trema (65)	*Prunus africana* (1)	*Ficus exasperata*	*Teclea nobilis*
*Alangium chinense* (54) (Pioneer)	Mimosaceae (48)	Alangium (54)		*Ficus mucuso*	
*Vernonia amygdalina* (39) - Colonizer	Compositae (Asteraceae) (41)	Vernonia (41)		*Ficus polita*	
*Bathedavia (javanica) (34)* Colonizer	Euphorbiaceae (33)	Albizia (38)		*Ficus sur*	
				*Ficus vallis-choudae*	
				*Khaya anthotheca*	
				*Lovoa trichilioides*	
				*Maesopsis eminii*	
				*Mangifera indica*	
				*Milicia exelsa*	
				*Olea welwitschii*	
				*Piptadeniastrum africanum*	
				*Prunus africana*	

During FGD discussions, participants named 39 tree species they could remember to have existed in the forest before it was severely degraded. Among the 39 mentioned species, 22 (56%) were observed during the forest inventory, while 17 species were not observed. (Appendix 3)

#### Population structure: diameter size class distribution

**Fig. 6:**
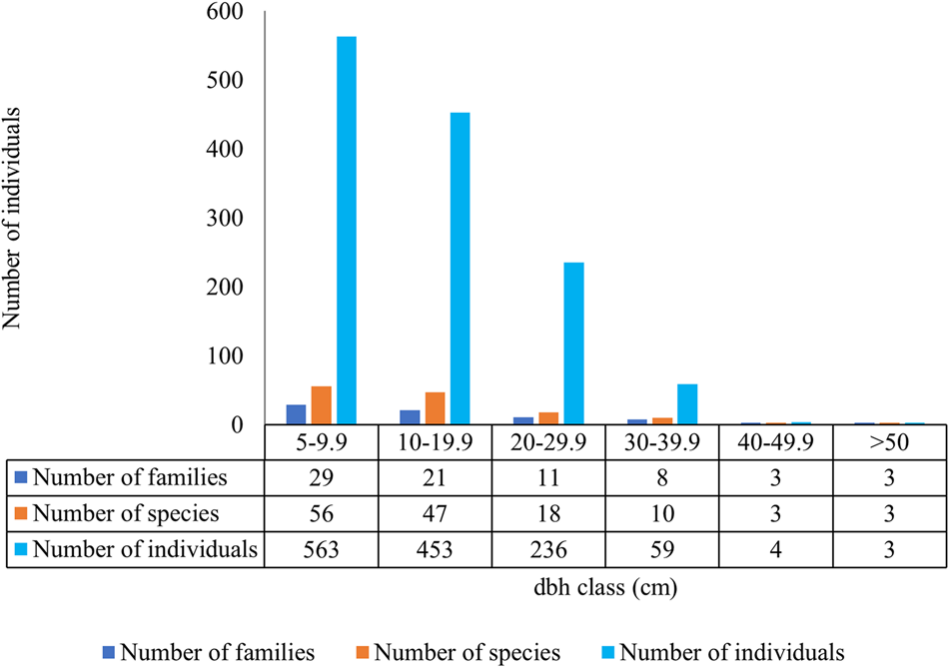
Diameter class distribution of individual trees, tree species and tree families in the Gangu biodiversity offset sites

The dominant diameter class of all the species encountered was 5–9.9 cm DBH, while the diameter class with the least number of tree stems was >50 cm DBH (Fig. 6). The historical native species dominate the lower diameter classes while the species used to restore the forest dominate the higher dominant diameter classes (Table 4).

**Table 4:** Diameter classes of dominant tree species in the Gangu biodiversity offset sites

Species	Tree stems	Diameter class (cm)					
		<10	10–19.9	20–29.9	30–39.9	40–49.9	>50
*Maesopsis eminii*	197	31	74	77	15		
*Cedrela odorata*	129	12	43	60	10	3	1
*Pseudospondias microcarpa*	121	75	44	1			1
*Terminalia superba*	113	31	26	38	17	1	
*Antiaris africana*	75	46	26	3			
*Trema orientalis*	73	22	24	22	4	1	
*Ficus sur*	59	25	28	5	1		
*Alangium chinensis*	54	37	17				
*Vernonia amygdalina*	39	31	7	1			
*Bathedavia (javanica)*	34	6	16	11	1		

### Regeneration: Extent to which the Offset is Providing Conditions for Regeneration

A total of 226 seedlings/resprouts (<5 cm DBH) were recorded in 2 m × 2 m 83 sampled subplots. The seedlings constitute 42 species, 39 genera, and 25 families (Appendix 2). The seedlings recorded in the offset sites were mainly dominated by native species, including *Blighia unijugate* (22.1%), *Ficus sp* (11.5%), *Aningeria altissima* (7.5%), *Maesa lanceolata* (6.2%), *Maesopsis eminii* (6.2%), *Antiaris africana* (5.8%), *Allophylus africanus* (3.1%), *Celtis africana* (3.1%), *Psidium cordatum* (3.1%), and *Terminalia ivorensis* (2.7%).

There were 7 species in the sampled plots represented by seedlings only (without trees above 5 cm DBH). These include: *Allophylus africanus*; *Citropsis articulata*; *Clausena anisata*: *Diospyros abyssinica*; *Phoenix reclinata*; *Syzygium guineense* and *Vangueria apiculata*.

## Discussion

The major challenges of restoration offsets highlighted in literature include uncertainty of restoration success and the large time-lags before degraded ecosystems can be restored to reference states (Maron et al. 2012; Bull et al. 2013). For the Gangu BO, restoration activities combined with protection resulted into increased forest cover, and enhancement of forest species composition in the BO sites after a period of 8 years. However, the permanence of the BO restoration outcomes is uncertain due to continued forest resource exploitation within offset sites by communities that have no alternative sources of livelihoods.

The forest cover analysis and social data reveal that restoration activities resulted in an increase in area under forest cover (THF-normal stock + THF-low stock) and reduction in area under bushland and farmland. In addition, restricted entry and resource access in the offset sites minimized human interference, especially the extent of farming and grazing, potentially increasing the survival rates of seedlings and sprouts in the offset sites. Studies elsewhere have shown that controlling livestock pressure is necessary for effective indigenous tree species regeneration (Wassie et al. 2009). Seedling survival rates are higher and seedlings grow faster because there is no browsing and trampling damage. Therefore, active restoration and management of the conservation areas can increase chances and rate of vegetation recovery in degraded areas. These findings are also in line with Curran et al.’s (2014) observation that active habitat restoration speeds up the restoration process.

The forest cover analysis also revealed a significant decline in THF-normal stock, which communities attribute to logging, farming practices, and weak law enforcement characterised by corruption. This implies that offset measures have not been effective in halting illegal forest resource extraction. Community dependence on the forest for resources especially firewood and charcoal was very high prior to the implementation of the offset. However, an attempt to address the loss of forest products through establishment of CFM arrangements did not comprehensively address matters of social equity. CFM arrangements that were meant to compensate for lost forest livelihoods as a result of BO implementation only considered directly compensating for lost cultivation land, leaving out other forest resource-user groups. The non-CFM members, who constituted the greatest proportion of the communities did not get alternative sources of forest products (Kigonya et al., Forthcoming). With monitoring and patrolling from both forest authorities and CFM members, continued logging usually in the wee-hours of the night depicts desperate need of fuel wood and lack of alternative livelihood sources among non-CFM members. These were restricted from obtaining firewood from the woodlots established by CFM members, which they have to buy, and is only obtained upon harvesting that is not frequent (Kigonya et al., Forthcoming). Therefore, offset design did not fully recognize the interdependence of the local communities on the forest ecosystem, and the necessity to reconcile local community values, resource demands, which is key for successful offset implementation and sustainability (World Bank 2020). The trees currently planted in the biodiversity offset sites are at a risk of being harvested by the communities when they mature, considering that there is evidence of increased degraded forest even when the sites are managed as a BO. An ecological assessment of other parts of Gangu CFR revealed a decrease in forest cover within the sites neighbouring the biodiversity offset sites (Kigonya et al., Forthcoming). Therefore, the establishment of the biodiversity offsetting sites can also cause leakage/perverse outcomes if community livelihood benefits are not adequately compensated for. High pressure on the forest for tree products threatens the permanence of the BO restoration benefits which is crucial for attaining net biodiversity benefits over time (Virah-Sawmy et al. 2014). The benefits are expected to be sustained during the commitment period and beyond for a successful BO (Calvet et al. 2019).

Since BOs are ideally supposed to compensate for loss of biodiversity elsewhere, the permanence of biodiversity benefits is paramount for legal and legitimacy purposes. The effectiveness of offsets is dependent on performance accountability that can be achieved through constant monitoring (Villarroya et al. 2014). Therefore, it is desired that BOs be managed in ways that minimize disturbances or impacts on the biodiversity components restored or conserved. Therefore, the capacity of the forest management to monitor the sites should be strengthened, while taking into consideration the customary rights of the offset adjacent communities. A combination of restoration and protection of offsets was recommended by Souza et al., (2021) to increase chances of restoration BO success. Considering that the Gangu CFR is a SNR, the management agency should enforce the restrictions of SNR, where access to and use of biodiversity components by the communities adjacent to the offset sites is regulated. To mitigate acts of bribery and corruption that could potentially compromise restoration success, there is need for a robust governance system that is trustworthy and transparent (Wainaina et al. 2021). While the NFA is directly financed by the government, institutional financial reports reveal that the budget allocations are low compared to what is needed to operationalise required forest management activities such as patrolling for illegal activities and resurveying and demarcating forest reserves (NFA 2016a; GoU 2021). There is thus need for additional financial support to ensure continued effective management of the offset sites. At a sector level, there is need to adhere to good forest governance principles such as carrying out regular governance assessments to ensure proper management, complying to the rule of law, transparency and low levels of corruption, and implementation of a coherent set of policies, laws and regulations both within the forest sector and other sectors that influence forest management (FAO 2012, 2018). There is also need to implement biodiversity-based livelihood compensations that benefit those impacted by offset measures, create alternative sources of products desired from the forests such as forest buffer zones close to the BO sites or community forests from which communities can continue obtaining forest resources, or widely distribute technologies that can lead to more efficient use of forest products such as energy saving cook stoves, to reduce demand and pressure on offset sites resources. The World Bank (2023) recommends that prior to implementation of projects, a census be carried out to identify persons and livelihoods that would be heavily affected by the projects. The results should then be the basis upon which livelihood restoration plans are developed to ensure that all of those heavily affected can have their livelihoods improved or restored.

Whereas the restoration activities involved planting of only 7 tree species, the study identified 62 tree species within the offset sites. Among these, 28 species had no seedlings, while 34 species were represented as both seedlings and trees. This indicates advanced regeneration for those species, and their seedlings are most likely to develop into mature trees. Among the species restored are singletons that were previously not reported in the forest ecosystem, nationally reserved/protected species (GoU 2016), as well as IUCN Red Listed species. Twenty two out of 39 tree species that were previously reported to have existed in the forest were also observed during the forest inventories. Out of the seven planted tree species, *Maesopsis eminii* and *Cedrela odorata* emerged among the most dominant tree species (first and second respectively). Although these two are not invasive in Uganda, they have been reported invasive elsewhere (Mwendwa et al. 2020; Van Der Meersch et al. 2021; Kilawe et al. 2022). It is important for future restoration BOs to take into consideration the potential negative interactions selected restoration species could have with local species. Six out of the 10 most dominant tree species were among the indigenous tree species that underwent natural regeneration.

The relatively high Shannon diversity index of 3.312 indicates that there is high species diversity and evenness within the BO sites. This is an indication of a stable plant community. An ecosystem with Shannon’s index values greater than 2 is regarded as medium to highly diverse in terms of species (Ortiz-Burgos 2016). The high Pieulous J evenness of 0.8025 is an indicator that the species in the offset sites have relatively similar abundance. This further confirms that after 8 years of BO implementation, many species have been restored through natural regeneration. The results are in line with Ssekuubwa et al’s., (2021) findings which indicate that passive restoration in tropical forests can recover forest composition within a relatively short period of time. The risk of the offset being dominated by a few planted species is not reflected in the current sites. Natural regeneration has been deemed highly effective and cost effective where soils have not been degraded and seed-dispersing fauna are present (Martínez‐Ramos et al. 2016; Brancalion and Chazdon 2017). However, in situations where communities are highly dependent on forest resources, active restoration will result into quick accumulation of biomass that can support community livelihoods within a short period of time (Crouzeilles et al. 2017).

Size-class distributions of trees in the study revealed that small diameter classes had large numbers of individuals and large diameter classes had small numbers of individuals. This depicts the classic “inverse J” shaped size class distribution curve indicating a stable and growing population (Condit et al. 1998). This indicates that the species are adapted, recruited fairly regularly, can propagate sustainably and constitute stable populations (Oliver and Larson 1996; Nduwayezu et al. 2015) and the low mortality of regenerating individuals.

Species regeneration could have mainly resulted from sprouting, an attribute that Mwavu and Witkowski (2008) reported as key to ensuring persistence of woody plant individuals and populations in tropical rain forests after selective timber, pole, and sapling harvesting. This could also indicate that the forest ecosystem had a large seed pool (Buschke and Sinclair 2019). The increase in the number of tree species through natural regrowth partly resulted from halting crop farming and grazing which hinders tree regeneration through uprooting and clearing of seedlings or sprouts, grazing, browsing, and trampling on the seedlings. Grazing has been reported in other studies to have significant negative impacts on germination, seedling survival, and growth (Jimenez et al. 2005; Wassie et al. 2009). The animals could have trampled or browsed on the seedlings, subsequently affecting their restoration and growth. The results also depict that it is only the trees that were planted as part of the restoration effort that have larger diameter size classes. The delayed germination and establishment of other indigenous trees could have resulted from permitted taungya farming, a forest management strategy in which trees are temporarily raised in association with agricultural crops in the first years of tree establishment until crop growth is impaired by tree canopy closure (Appiah et al. 2020). Although taungya farming reduces the costs of forest rehabilitation, as the farmers are assumed to tend the trees, as they benefit through planting their own crops (Fatma et al. 2020), the practice may have constrained the possibility of seedling growth prior to canopy closure of the planted trees because farmers cleared them when weeding their crops and only preserved the planted tree species that the NFA staff were more concerned about.

## Conclusion

Our results provide empirical evidence on the potential of restoration offsets to recover forest vegetation, high species composition, evenness, and diversity of tropical forests in a relatively short period of time. The study also shows that combining restoration activities with measures that limit further forest disturbances such as regulating forest resource access and use through patrols (by both forest authorities and communities through collaborative forest arrangements) and providing alternative sources of forest resources to adjacent forest communities, can enhance restoration offset outcomes. Reduced human activities or disturbances including eliminating Taungya farming during the initial years of restoration in offset sites could potentially lead to higher regenerating survival rates and shorter period for complete forest recovery. However, lost biodiversity-based livelihoods of all those impacted by offset implementation ought to be compensated to possibly secure compliance with offset access and use regulations and minimize illegal resource extraction activities.

### Supplementary Information

Appendices
