# Supplementary material for: “Biodiversity Offsetting” in Uganda’s Protected Areas: A Pathway to Restoration of Forest Biodiversity?
Source: Environ Manage. 2024 May 10;73(6):1134–49. doi: 10.1007/s00267-024-01982-6 (PMC11136839; doi:10.1007/s00267-024-01982-6)
Supplement: Supplementary file 1 — Appendices [file 267_2024_1982_MOESM1_ESM.docx]

**“Biodiversity offsetting” in Uganda’s protected areas: A pathway to restoration of forest biodiversity?**

Environmental Management

Ritah Kigonya^a^*, Patrick Byakagaba^b^, Edward Ssenyonjo^c^ and Charlotte Nakakaawa Jjunju^a^

*corresponding author: Ritah Kigonya; Department of Geography, NTNU, Edvard Bulls Veg 1, 7491, Trondheim, Norway; Email: [ritah.kigonya@ntnu.no](mailto:ritah.kigonya@ntnu.no)

**Appendix 1: Areas of forests cleared in individual forest reserves during the establishment of the Kawanda-Masaka electricity transmission line**

| **Name of Forest Reserve** | **Length of wayleaves through the reserve (Km)** | **Area of forest cleared (Ha)** |
| --- | --- | --- |
| Lwamunda CFR | 0.76 | 3.04 |
| Lufuka CFR | 1.07 | 4.28 |
| Kyansozi CFR | 1.18 | 4.72 |
| **Gangu CFR** | **1.76** | **7.04** |
| Kalandazi CFR | 0.85 | 3.40 |
| Buwa CFR | 0.79 | 3.16 |
| Nabijoka LFR | 0.37 | 1.48 |
| **TOTAL** | **6.78** | **27.12** |

Source: SMEC International Pty Ltd, (2010); In bold is the study area for this paper

**Appendix 2: Summary of species, genera and families**

| **Species** | **Genus** | **Family** | **No of trees (>5cm dbh)** | **No of seedlings/sprouts** |
| --- | --- | --- | --- | --- |
| *Alangium chinense* | Alangium | Cornaceae | 54 | 2 |
| *Albizia coriaria* | Albizia | Mimosaceae | 1 |  |
| *Albizia grandibracteata* | Albizia | Mimosaceae | 18 |  |
| *Albizia zygia* | Albizia | Mimosaceae | 19 | 1 |
| *Alchornea sp* | Alchornea | Euphorbiaceae | 1 |  |
| *Aningeria altissima* | Aningeria | Sapotaceae | 3 | 17 |
| *Anthocleista grandiflora* | Anthocleista | Gentianaceae | 2 |  |
| *Antiaris africana* | Antiaris | Moraceae | 71 | 13 |
| *Artocarpus heterophyllus* | Artocarpus | Moraceae | 9 | 4 |
| *Bathedavia* | Bathedavia | Unkown | 34 |  |
| *Blighia unijugata* | Blighia | Sapindaceae | 29 | 50 |
| *Bosqueia phoberos* | Bosqueia | Moraceae | 10 | 1 |
| *Bridelia micrantha* | Bridelia | Euphorbiaceae | 1 | 3 |
| *Canarium schweinfurthii* | Canarium | Burseraceae | 7 | 1 |
| *Canthium vulgare* | Canthium | Rubiaceae | 7 | 4 |
| *Cedrela odorata* | Cedrela | Meliaceae | 127 | 1 |
| *Celtis africana* | Celtis | Ulmaceae | 24 | 7 |
| *Celtis zenkeri* | Celtis | Ulmaceae | 3 |  |
| *Chaetachme aristata* | Chaetachme | Ulmaceae | 15 | 2 |
| *Coloncoba* | Unknown | Unkown | 1 |  |
| *Ekebergia capensis* | Ekebergia | Meliaceae | 2 |  |
| *Entandrophragma utile* | Entandrophragma | Meliaceae | 12 |  |
| *Fagara macrophylla* | Fagara | Rutaceae | 1 |  |
| *Fagaropsis angolensis* | Fagaropsis | Rutaceae | 6 |  |
| *Ficus exasperata* | Ficus | Moraceae | 2 | 1 |
| *Ficus mucuso* | Ficus | Moraceae | 26 | 1 |
| *Ficus polita* | Ficus | Moraceae | 3 |  |
| *Ficus sur* | Ficus | Moraceae | 59 |  |
| *Ficus vallis-choudae* | Ficus | Moraceae | 14 |  |
| *Funtumia elastica* | Funtumia | Apocynaceae | 26 | 5 |
| *Harungana madagascariensis* | Harungana | Guttiferae | 1 |  |
| *Khaya anthotheca* | Khaya | Meliaceae | 18 |  |
| *Lovoa trichilioides* | Lovoa | Meliaceae | 10 |  |
| *Macaranga schweinfurthii* | Macaranga | Euphorbiaceae | 13 | 2 |
| *Maesa lanceolata* | Maesa | Myrsinaceae | 5 | 14 |
| *Maesopsis eminii* | Maesopsis | Rhamnaceae | 174 | 14 |
| *Mangifera indica* | Mangifera | Anacardiaceae | 2 |  |
| *Margaritaria discoideus* | Margaritaria | Euphorbiaceae | 14 | 1 |
| *Markhamia lutea* | Markhamia | Bignoniaceae | 16 | 1 |
| *Milicia excelsa* | Milicia | Moraceae | 4 |  |
| *Monodora myristica* | Monodora | Annonaceae | 6 |  |
| *Morinda lucida* | Morinda | Rubiaceae | 8 |  |
| *Olea welwitschii* | Olea | Oleaceae | 5 |  |
| *Persea americana* | Persea | Lauraceae | 5 |  |
| *Piptadeniastrum africanum* | Piptadeniastrum | Mimosaceae | 9 |  |
| *Prunus africana* | Prunus | Rosaceae | 1 |  |
| *Pseudospondias microcarpa* | Pseudospondias | Anacardiaceae | 121 | 4 |
| *Psidium cordatum* | Psidium | Myrtaceae | 2 | 7 |
| *Pycnanthus angolensis* | Pycnanthus | Myristicaceae | 8 | 1 |
| *Sapium ellipticum* | Sapium | Euphorbiaceae | 4 | 1 |
| *Senna spectabilis* | Senna | Caesalpiniaceae | 5 | 4 |
| *Spathodea campanulata* | Spathodea | Bignoniaceae | 14 | 2 |
| *Sterculia dawei* | Sterculia | Sterculiaceae | 32 | 1 |
| *Teclea nobilis* | Teclea | Rutaceae | 1 | 2 |
| *Terminalia ivorensis* | Terminalia | Combretaceae | 16 | 6 |
| *Terminalia superba* | Terminalia | Combretaceae | 111 | 1 |
| *Tetrapleura tetraptera* | Tetrapleura | Mimosaceae | 1 |  |
| *Trema orientalis* | Trema | Ulmaceae | 65 |  |
| *Trichilia dregeana* | Trichilia | Meliaceae | 3 |  |
| *Trichilia emetica* | Trichilia | Meliaceae | 16 | 4 |
| *Vernonia amygdalina* | Vernonia | Compositae (Asteraceae) | 39 | 2 |
| *Vernonia auriculifera* | Vernonia | Compositae (Asteraceae) | 2 |  |
| *Allophylus africanus* |  |  |  | 7 |
| *Citropsis articulata* |  |  |  | 1 |
| *Clausena anisata* |  |  |  | 1 |
| *Diospyros abyssinica* |  |  |  | 3 |
| *Ficus sp* |  |  |  | 26 |
| *Phoenix reclinata* |  |  |  | 1 |
| *Syzygium guineense* |  |  |  | 2 |
| *Vangueria apiculata* |  |  |  | 4 |
| Unknown seedling |  |  |  | 1 |

**Appendix 3 Common tree species in Gangu forest prior to the degradation of the forest.**

| **Appeared in forest inventory** | **Did not appear in forest inventory** |
| --- | --- |
| *Albizia coriaria* | *Alstonia boonei* |
| *Albizia grandibracteata* | *Antiaris toxicaria* |
| *Albizia zygia* | *Beilschmiedia ugandensis* |
| *Alchornea cordifolia* | *Celtis mildbraedii* |
| *Aningeria altissima* | *Entada abyssinica* |
| *Artocarpus heterophyllus* | *Erythrina abyssinica* |
| *Blighia unijugata* | *Ficus glumosa* |
| *Bosqueia phoberos* | *Ficus natalensis* |
| *Canarium schweinfurthii* | *Ficus ovata (F. brachypoda)* |
| *Ficus exasperata* | *Hallea rubrostipulata* |
| *Ficus mucuso* | *Hallea stipulosa* |
| *Funtumia elastica* | *Neoboutonia macrocalyx* |
| *Lovoa trichilioides* | *Polyscias fulva* |
| *Maesopsis eminii* | *Ricinus communis* |
| *Margaritaria discoideus* | *Saba comorensis* |
| *Milicia excelsa* | *Solanum gigantum* |
| *Piptadeniastrum africanum* | *Zanthoxylum gilletii* |
| *Prunus africana* |  |
| *Pseudospondias microcarpa* |  |
| *Teclea nobilis* |  |
| *Trema orientalis* |  |
| *Vernonia amygdalina* |  |
